# Diagnostic Value of Cardiac Magnetic Resonance Imaging (CMRI) in the Evaluation of Arrhythmia and Cardiomyopathy

**DOI:** 10.7759/cureus.72675

**Published:** 2024-10-30

**Authors:** RP Singh, Preeti Singh, TS Kler, Shoma Mukerjee Sharma

**Affiliations:** 1 Cardiology, Hind Institute of Medical Sciences, Sitapur, IND; 2 Cardiology, Pushpawati Singhania Research Institute (PSRI) Hospital, New Delhi, IND; 3 Prosthodontics, Saraswati Dental College, Lucknow, IND; 4 Prosthodontics, Pushpawati Singhania Research Institute (PSRI) Hospital, New Delhi, IND; 5 Cardiology, B. L. Kapur (BLK) - Max Hospital, New Delhi, IND; 6 Radiology, Mahajan Imaging, Fortis Hospital, New Delhi, IND; 7 Radiology, Pushpawati Singhania Research Institute (PSRI) Hospital, New Delhi, IND

**Keywords:** arrhythmias, cad: coronary artery disease, cardiac mri (cmr), cardiomyopathy, late gadolinium enhancement, lv dysfunction

## Abstract

Background

Cardiac magnetic resonance imaging (CMRI) is now the gold standard noninvasive method for evaluating ventricular volume, mass, and ejection fraction - crucial indicators for diagnosing and prognosticating heart failure. Renowned for its accuracy and reliability, CMRI utilizes advanced techniques, such as late gadolinium enhancement and T1 and T2 mapping, to produce three-dimensional images with high spatial and temporal resolution, all without ionizing radiation. One of CMRI’s key strengths lies in its capacity to characterize myocardial tissue, providing valuable diagnostic and prognostic insights into underlying cardiac conditions. Recent studies underscore CMRI’s feasibility, safety, and emerging cost-effectiveness in selected clinical settings. However, the complex etiologies of arrhythmias and cardiomyopathies, marked by varied structural and functional myocardial changes, continue to present diagnostic challenges. While CMRI’s role is expanding, comprehensive observational studies are essential to fully elucidate its diagnostic utility in these cases.

Aim

This study aims to assess the utility of CMRI in the etiological diagnosis of heart failure and arrhythmias, addressing current knowledge gaps and contributing to clinical practice insights. Overall, CMRI is highlighted as a versatile and effective imaging modality for evaluating diverse aspects of cardiac health.

Methods

This single-center, hospital-based, non-randomized, prospective observational study was conducted in the cardiology department of Pushpawati Singhania Research Institute (PSRI) Hospital in New Delhi, India, from December 2019 to December 2021. It included patients presenting with arrhythmias, cardiomyopathies, unexplained left ventricular (LV) dysfunction, suspected arrhythmogenic right ventricular dysplasia, myocarditis, hypertrophic cardiomyopathy, as well as infiltrative and granulomatous diseases. Only patients who provided consent for cardiac MRI were included. Cardiac MRI tests were performed and analyzed for all participants to evaluate their diagnostic utility across these conditions.

Results

The study involved 100 participants, with 75% diagnosed with cardiomyopathy and 25% with arrhythmia. Among the cardiomyopathy cases, 24 patients (32%) had ischemic cardiomyopathy, while 51 patients (68%) had nonischemic cardiomyopathy. LV dysfunction was noted in 65 patients (86.6%), with a viable scar present in three patients (4%) and myocarditis diagnosed in four patients (5.3%). Chamber dilatation was observed in 13 patients (52%). In the arrhythmia group, six patients (24%) exhibited prolonged native T1, while eight patients (32%) had normal cardiac MRI results. These findings highlight the significant utility of cardiac MRI in diagnosing and characterizing various cardiac conditions within the study population.

Conclusions

The study found that cardiac MRI exhibits a strong positive predictive value in diagnosing various types of cardiomyopathies. This suggests that when CMRI identifies cardiomyopathy, it is likely to provide an accurate diagnosis, thereby enhancing its utility in clinical practice for effective patient management and treatment planning.

## Introduction

The paper emphasizes the growing significance of cardiac magnetic resonance imaging (CMRI) in diagnosing and characterizing various heart conditions, particularly cardiomyopathies and arrhythmias. It highlights CMRI’s capability to provide detailed insights into heart structure, function, and tissue characteristics, which are essential for accurate diagnosis and treatment planning. CMRI shows a strong positive predictive value in diagnosing different types of cardiomyopathies, especially when used alongside techniques like late gadolinium enhancement (LGE). It effectively detects myocarditis by identifying characteristic LGE patterns and elevated native T1 values, which indicate myocardial inflammation. Additionally, CMRI excels at differentiating between ischemic and nonischemic cardiomyopathies by analyzing LGE patterns and assessing myocardial tissue health, which is crucial for determining the underlying causes of heart dysfunction. While CMRI’s role in assessing arrhythmias is still evolving, it can help identify structural abnormalities that may contribute to these conditions.

CMRI is highlighted for its accuracy and reliability, particularly with advanced imaging methods like LGE and T1/T2 mapping [1]. Furthermore, CMRI provides high-resolution, three-dimensional imaging without the risks associated with ionizing radiation [2]. One of its major advantages is its ability to characterize myocardial tissue, enhancing both diagnostic and prognostic capabilities in identifying heart conditions [3]. Recent studies suggest that CMRI is safe, feasible, and potentially cost-effective for specific clinical indications [4]. However, arrhythmias and cardiomyopathies present diagnostic challenges due to their varied causes and manifestations, highlighting the need for further observational studies to clarify CMRI’s diagnostic value in these contexts [5].

## Materials and methods

Study design and population

The present study was a single-center, hospital-based, prospective, non-randomized observational research conducted in the Department of Cardiology of Pushpawati Singhania Research Institute (PSRI) Hospital in New Delhi, India, from December 2019 to December 2021. The study received approval from the institutional ethics committee, and written informed consent was obtained from all participants before enrollment.

The inclusion criteria were as follows: (i) patients with arrhythmia; (ii) patients with cardiomyopathies, specifically unexplained left ventricular (LV) dysfunction (defined as an LV ejection fraction <50%); (iii) suspected arrhythmogenic right ventricular dysplasia (ARVD); (iv) suspected myocarditis; (v) suspected hypertrophic cardiomyopathy (HCM); and (vi) infiltrative and granulomatous diseases. Additionally, participants needed to be willing to undergo magnetic resonance imaging (MRI) and consent to participate in the study.

Exclusion criteria included moribund patients, individuals with severe claustrophobia, those experiencing severe multi-organ dysfunction, patients with ferromagnetic implants such as central nervous system aneurysmal clips, individuals with MRI-incompatible implants, and those with severe renal dysfunction.

Methodology

Patients were informed about the cardiac MRI procedure in advance and instructed to arrive at the center after fasting for at least 12 hours. Peripheral IV access was established, and patients’ medication histories were reviewed. For adenosine infusion, two separate IV accesses were required, preferably in different arms - one for adenosine and the other for the contrast agent. The contrast injection protocol involved administering gadolinium at a dose of 0.05-0.1 mmol/kg, followed by a saline chasing bolus of 30 mL. The adenosine infusion was administered at a rate of 140 micrograms/kg for two to four minutes.

The study was conducted with patients in the supine position using a Philips Prodiva 1.5 T MRI scanner, equipped with a high-resolution digital coil and cardiac analysis software. T1-weighted double inversion recovery fast spin echo (FSE) and T2-weighted triple inversion recovery FSE sequences were acquired in axial planes for structural assessment and tissue characterization. High temporal resolution images were obtained throughout the cardiac cycle with electrocardiographic gating, allowing for the identification of end-systole and end-diastole. A steady-state free precession sequence was performed for functional assessment, acquiring short-axis four-chamber and two-chamber stacks.

Gadolinium chelate was infused intravenously via a peripheral IV line. Phase-sensitive inversion recovery (PSIR) sequences were acquired 10 minutes after gadolinium infusion to evaluate LGE, preceded by a TI scout to determine the optimal nulling time of the LV myocardium. PSIR sequences were repeated with phase swapping to optimize the evaluation of fibrosis and scarring. For stress cardiac MRI, adenosine was administered intravenously, and magnetic resonance images were acquired at the peak of stress.

Statistical analysis

Statistical analysis was conducted using IBM SPSS Statistics for Windows, Version 25.0 (Released 2017; IBM Corp., Armonk, NY, USA). Variables were presented as means and standard deviations, as well as frequency counts and percentages.

## Results

A total of 100 patients with heart failure or arrhythmia were included in the study. Among these, 75 patients were diagnosed with cardiomyopathy, while 25 patients presented with arrhythmia. The cardiomyopathy group was further subdivided into ischemic cardiomyopathy, which included 24 patients (32%), and nonischemic cardiomyopathy, which comprised 51 patients (68%). The baseline clinical characteristics of patients with cardiomyopathy are presented in Table 1.

**Table 1:** Baseline clinical characteristics of patients with cardiomyopathy (n = 75) ARVD: arrhythmogenic right ventricular dysplasia; HCM: hypertrophic cardiomyopathy; LV: left ventricular

Clinical characteristics	n (%)
LV dysfunction/heart failure	65 (86.6%)
Viability/scar assessment	3 (4%)
Suspected myocarditis	4 (5.3%)
Suspected ARVD	1 (1.3%)
Suspected HCM	2 (2.6%)

The mean age of the patient population was 52.7 ± 16.1 years. Within the cardiomyopathy group, 65 patients (86.6%) presented with LV dysfunction or heart failure, while three patients (4%) underwent viability/scar assessment, four patients (5.3%) were diagnosed with myocarditis, one patient (1.3%) had ARVD, and two patients (2.6%) were identified with HCM. Among the subset of cardiomyopathies, 67 patients (89.3%) exhibited LV dysfunction. Specifically, LV dysfunction was observed in 46 patients (90.2%) within the nonischemic cardiomyopathy subgroup and in 21 patients (87.5%) within the ischemic cardiomyopathy subgroup. Coronary angiography was performed on 62 patients, revealing significant triple vessel disease in 15 patients, significant double vessel disease in one patient, and single vessel disease in 16 patients, of which eight patients had an obstruction of less than 50% in the involved vessel. The coronary angiographic findings were normal in 32 patients, and coronary angiography was not conducted in 13 patients. Clinical characteristics and coronary angiography results for patients with cardiomyopathy are outlined in Table 2.

**Table 2:** Coronary angiography findings in patients with cardiomyopathy (n = 75) CAG: coronary angiography; DVD: double vessel disease; SVD: single vessel disease; TVD: triple vessel disease

CAG findings	Nonischemic cardiomyopathy (n = 51)	Ischemic cardiomyopathy (n = 24)
SVD	1 (1.9%)	5 (20.8%)
Non-critical SVD	7 (13.7%)	1 (4.2%)
DVD	0 (0%)	1 (4.2%)
TVD	0(0%)	15 (62.5%)
Normal CAG	31(60.8%)	1 (4.2%)
CAG not done	12 (23.5%)	1 (4.2%)

Ischemic cardiomyopathy was identified in 24 patients (32%) within the cardiomyopathy group. The LGE pattern was predominantly subendocardial in 20 patients (80%) in the ischemic cardiomyopathy subgroup, while five patients (20%) exhibited myocardial edema along with prolonged native T1 values; however, no LGE uptake was detected. Nonischemic cardiomyopathy was present in 51 patients (68%) of the cardiomyopathy group, among whom 26 patients (51%) were diagnosed with idiopathic dilated cardiomyopathy (DCM) and 25 patients (49%) had alternative diagnoses, including myocarditis, infiltrative disorders, HCM, and LV non-compaction. The CMRI findings for patients with cardiomyopathy are illustrated in Table 3.

**Table 3:** Cardiac MRI findings in patients with cardiomyopathy (n = 75) ARVD: arrhythmogenic right ventricular dysplasia; DCM: dilated cardiomyopathy; HCM: hypertrophic cardiomyopathy; LVNC: left ventricle non-compaction; MRI: magnetic resonance imaging

Final outcome	n = 75 (%)
Ischemic cardiomyopathy	24 (32%)
Nonischemic cardiomyopathy	51 (68%)
Cardiac MRI-based outcome among nonischemic cardiomyopathy	n = 51
DCM	26 (50.9%)
Myocarditis	07 (13.7%)
Infiltrative disorder	06 (11.8%)
HCM	05 (9.8%)
ARVD	01 (1.9%)
LVNC	03 (5.9%)
Normal cardiac MRI	03 (5.9%)

**Figure 1:**
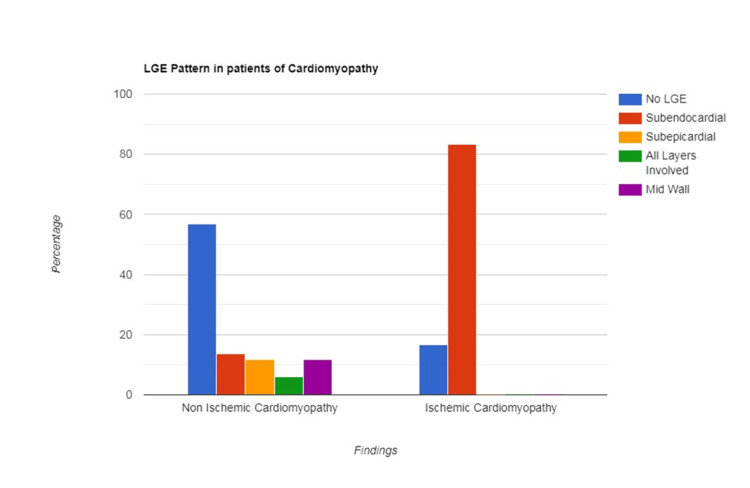
Different patterns of LGE LGE: late gadolinium enhancement

Among the 26 patients diagnosed with DCM, 20 patients (76.9%) exhibited LGE-negative myocardium. The LGE-negative myocardium demonstrated significantly higher native T1 values in 13 patients (65%), while one patient (4%) showed focal LGE uptake. Myocarditis was identified in a total of eight patients (15.7%). In these myocarditis patients, LGE was predominantly localized to the lateral and inferior walls, with the most common pattern being mid-wall in 23 patients (87.5%) and subepicardial in three patients (12.5%). Notably, native T1 values were significantly elevated in all patients diagnosed with myocarditis. Different LGE patterns among various cardiomyopathies are illustrated in Table 4 and Figure 1.

**Table 4:** Different LGE patterns among cardiomyopathy ICM: ischemic cardiomyopathy; LGE: late gadolinium enhancement; NICM: nonischemic cardiomyopathy

	LGE pattern in NICM (n = 51)	LGE pattern in ICM (N = 24)	p-value
Subendocardial	7 (13.7%)	20 (83.3%)	<0.001
Mid wall	6 (11.8%)	0 (0%)	
Subepicardial	6 (11.8%)	0 (0%)	
All layers involved	3 (5.8%)	0 (0%)	
No LGE	29 (56.9%)	4 (16.7%)	

In this study, a total of 25 patients were diagnosed with arrhythmia. The mean age of these patients was 46.28 ± 16.84 years, with a predominance of males (84%). Ventricular ectopic arrhythmia was present in eight patients (32%), while ventricular tachycardia was observed in six patients (24%). Non-sustained ventricular tachycardia was noted in one patient (4%), supraventricular ectopy in four patients (16%), supraventricular tachycardia in three patients (12%), atrial fibrillation in two patients (8%), and atrial flutter in one patient (4%).

CMRI findings revealed that one patient (4%) had ischemic cardiomyopathy, one patient (4%) had arrhythmogenic right ventricular cardiomyopathy, and one patient (4%) was diagnosed with myocarditis. Additionally, two patients (8%) were identified with HCM, two patients (8%) had persistent left superior vena cava drainage, and two patients (8%) were diagnosed with valvular heart diseases. Six patients (24%) showed abnormal CMRI results without a definitive diagnosis, while eight patients (32%) had normal CMRI findings despite the presence of arrhythmia.

LV dysfunction was observed in five patients (20%), right ventricular dysfunction was noted in five patients (20%), and chamber dilatation was present in 13 patients (52%). Wall edema was seen in one patient (4%), who initially presented with ventricular tachycardia and was ultimately diagnosed with myocarditis. Prolonged native T1 values were recorded in six patients (24%), while prolonged T2 values were noted in only two patients (8%). LGE enhancement was detected in two patients (8%) with ventricular tachycardia. Clinical characteristics and CMRI findings among patients with arrhythmia are detailed in Table 5.

**Table 5:** Clinical characteristics and CMRI findings among patients with arrhythmia (n = 25) ARVC: arrhythmogenic right ventricular cardiomyopathy; CMRI: cardiac magnetic resonance imaging; HCM: hypertrophic cardiomyopathy; ICM: ischemic cardiomyopathy; LGE: late gadolinium enhancement; LSVC: left superior vena cava draining; LV: left ventricle; MRI: magnetic resonance imaging; NSVT: non-sustained ventricular tachycardia; RV: right ventricle

Arrhythmia	n (%)
Types of arrhythmias	
Frequent ventricular ectopic	8 (32%)
Ventricular tachycardia	6 (24%)
NSVT	1 (4%)
Supraventricular ectopic	4 (16%)
Supraventricular tachycardia	3 (12%)
Atrial fibrillation	2 (8%)
Atrial flutter	1 (4%)
Cardiac MRI findings	
ICM	1 (4%)
ARVC	1 (4%)
Myocarditis	2 (8%)
HCM	2 (8%)
Persistent LSVC	2 (8%)
Valvular heart diseases	2 (8%)
Abnormal Cardiac MRI without definitive diagnosis	6 (24%)
Normal cardiac MRI	8 (32%)
LV dysfunction	5 (20%)
RV dysfunction	5 (20%)
Chamber dilatation	13 (52%)
Wall edema	1 (4%)
Prolonged native T1	6 (24%)
Prolonged native T2 was prolonged	2 (8%)
LGE	2 (8%)

## Discussion

CMRI has rapidly evolved in recent years and is regarded as a reliable gold standard for assessing cardiac function. LGE CMRI serves as an effective noninvasive imaging modality for diagnosing cardiomyopathy and evaluating the patterns and degrees of myocardial enhancement in both ischemic and nonischemic cardiomyopathies [6]. Furthermore, CMRI enables accurate assessment of myocardial anatomy, function, perfusion, and pathology in a noninvasive manner [6]. Previous research by Kanagala et al. demonstrated that CMRI could identify previously undetected alterations in a significant number of patients with heart failure [1,7].

In their study, Jackson et al. noted that myocardial hyperenhancement, indicative of significant myocardial scarring, is consistently observed in patients with chronic ischemic cardiomyopathy, while it is relatively uncommon in those with idiopathic DCM [8]. In ischemic conditions, hyperenhancement typically presents as a subendocardial pattern, while mid-wall enhancement, associated with fibrosis, suggests a nonischemic origin. The severity of functional abnormalities in these patients correlates with the extent of myocardial enhancement. Additionally, focal myocardial enhancement accompanied by wall motion abnormalities strongly correlates with active myocarditis as defined by histopathological criteria. In cases of myocarditis, hyperenhancement typically exhibits a “nonischemic” pattern, affecting the epicardial quartile of the ventricular wall. This differential presentation underscores the importance of CMRI in distinguishing between ischemic and nonischemic myocardial diseases, thereby enhancing diagnostic accuracy and guiding clinical management.

In our study, normal CMRI findings were observed in patients with arrhythmia. Identifying the underlying arrhythmic substrate is crucial for effective planning, treatment, and prognosis. However, CMRI can be challenging in patients with irregular heart rates. A significant proportion of patients may display elements of underlying heart disease on CMRI, even when other imaging modalities, including echocardiography, yield normal results [9]. Delayed contrast-enhanced CMRI is useful for noninvasive tissue characterization, revealing distinct patterns of hyperenhancement in ischemic and nonischemic cardiomyopathies [10]. The presence of regional myocardial fibrosis can be readily detected using LGE, while diffuse myocardial fibrosis can be assessed through parametric mapping, enhancing our understanding of the underlying etiology of arrhythmia. A normal CMRI scan offers reassurance for most patients, allowing clinicians to redirect their focus toward unrelated causes of arrhythmia [5].

The limitations of CMRI include its availability, the need for specialized expertise, financial costs, and exclusion criteria for patients with non-MR-compatible devices, cerebrovascular clips, or metallic objects in the eye. Additionally, patients who experience significant breathlessness or claustrophobia may be unable to undergo scanning [11]. The modified 2018 Lake Louise Criteria for myocarditis include (1) edema assessed by T2 imaging and (2) myocardial injury evaluated by T1 imaging. In patients with myocarditis, native T1 values are significantly elevated due to myocardial inflammation and edema [12]. The LGE pattern associated with myocarditis is primarily localized to the lateral and inferior walls, with the predominant patterns being mid-wall (84.4%) and subepicardial in nature [13]. The presence of LGE in the mid-wall and subepicardial regions of the left ventricle is characteristic of viral myocarditis and has been validated against histological findings [14].

In our study, a total of eight patients (15.7%) were diagnosed with myocarditis, with LGE predominantly affecting the lateral and inferior walls. The predominant patterns of LGE were mid-wall (87.5%) and subepicardial (12.5%). Our findings indicated significantly increased native T1 values in all patients with myocarditis (100%), aligning with the results from other studies referenced above.

Limitations

This study identified certain limitations in utilizing CMRI for diagnosing arrhythmia, potentially due to the small sample size and the inclusion of both ventricular and atrial arrhythmias. Additionally, the study acknowledges that the small sample size, single-center design, and limited findings within the arrhythmia group may influence the generalizability of the results.

## Conclusions

Despite its limitations, the research underscores the value of CMRI as a powerful diagnostic tool in cardiology. Its capability to deliver comprehensive insights into heart health renders it invaluable for effective patient management and treatment planning. This research paper provides substantial evidence supporting the use of CMRI in evaluating complex cardiac conditions. By accurately identifying the underlying causes of heart disease, CMRI enables clinicians to make informed decisions and implement personalized treatment strategies, ultimately leading to improved patient outcomes.
